# Hydroponics and elicitation, a combined approach to enhance the production of designer secondary medicinal metabolites in *Silybum marianum*

**DOI:** 10.3389/fpls.2022.897795

**Published:** 2022-08-10

**Authors:** Bismillah Mubeen, Ammarah Hasnain, Riffat Mehboob, Rabia Rasool, Ayesha Riaz, Shymaa Abdelsattar Elaskary, Muhammad Muntazir Shah, Tallat Anwar Faridi, Inam Ullah

**Affiliations:** ^1^Institute of Molecular Biology and Biotechnology, The University of Lahore, Lahore, Pakistan; ^2^Lahore Medical Research Centre, LLP and LMRC Laboratories, Lahore, Pakistan; ^3^Department of Zoology, GC Women University, Faisalabad, Pakistan; ^4^Medical Microbiology and Immunology, Faculty of Medicine, Menoufia University, Al Minufiyah, Egypt; ^5^Medical Microbiology and Immunology, Faculty of Medicine, King Abdulaziz University, Rabigh, Saudi Arabia; ^6^Punjab Forensic Science Agency, Lahore, Pakistan; ^7^University Institute of Public Health, Faculty of Allied Health Sciences, The University of Lahore, Lahore, Paksitan

**Keywords:** *Silybum marianum*, hydroponic culture, elicitation, *Aspergillus niger*, methyl jasmonate, silver nanoparticles, superoxide dismutase

## Abstract

Medicinal plants have been used to cure human diseases since decades. *Silybum marianum*, a medicinal plant, is regarded as a source of secondary metabolites with therapeutic value against liver diseases and diabetes. The present study was conducted to enrich the production of secondary metabolites in the vegetative parts of *Silybum marianum* using elicitation strategy in hydroponic system with different elicitors. The elicitors of fungus *Aspergillus niger* (0.2 g/L), methyl jasmonate (MeJA) (100 μM) and silver nanoparticles (AgNPs) (1 ppm) were added in hydroponic medium, individually and in combination form to the 15 days old plant. The elicitor-treated plants were harvested at different time points (24–144 h; increment 24 h) and their biochemical parameters like phenolics, flavonoids, nitric oxide (NO), and superoxide dismutase (SOD) were analyzed. The results showed hyper-accumulation of these biochemical contents, especially in response to MeJA (100 μM), followed by AgNPs (1 ppm) and co-treatment of AgNPs (1 ppm) with other elicitors. The results revealed that the treatment with MeJA (100 μM) exhibited the highest flavonoid (304 μg g^–1^), phenolic (372 μg g^–1^), and SOD (16.2 U g^–1^) contents. For NO levels, the maximum value of 198.6 nmole g^–1^ was achieved in response to the treatment with MeJA + Green synthesized AgNPs (100 μM + 1 ppm). Our findings depicted an enhanced production of medicinally important plant secondary metabolites and antioxidants; hence, the method applied in this study can play a significant role to improve therapeutic values of the plants.

## Introduction

*Silybum marianum* (milk thistle), a natural herb, is a rich source of medicinally important antioxidant biocompounds such as phenols. These natural compounds are known for their medicinal properties particularly for anticancerous activity (Shah et al., 2020). *S. marianum* is a major source of medicinally important secondary metabolites; however, problems pertaining to its low production due to seasonal, geographic or environmental changes remain a challenge (Mosihuzzaman, 2012; Shah et al., 2020). Therefore, there is a need to develop systems to elicit the production of bioactive compounds of medicinal importance specifically in the vegetative parts of the plant. *S. marianum* possesses a great commercial value and in order to maintain its huge market demand continuous efforts have been made in the field cultivation of this medicinal herb (Isah et al., 2018). Unfortunately, the secondary metabolite content of field-grown *S. marianum* is significantly affected by genetic, physiological, ecological and environmental factors (Maggini et al., 2014; Mahanom, 2018). All these factors lead to qualitative and quantitative changes in commercially produced pharmaceutical preparations of *S. marianum*. Therefore, in order to improve its yield, there is a need to establish a protocol for culturing *S. marianum* under *in vitro* conditions to yield standardized concentrations of biologically active compounds (Graf et al., 2007; Bekheet, 2011). A lot of experimental work has been conducted to achieve higher yields in the production system, and alterations in composition of culture media are the most common methods used in recent decades (Del Campo et al., 2007; Weathers et al., 2010).

There are several methods to improve secondary metabolite production, including changes in environmental conditions, use of elicitors or precursors and biotransformation (Ruiz-García and Gómez-Plaza, 2013; Liu et al., 2019). Among these methods, elicitation is one of the effective techniques which activates the defense mechanisms of plants (Baenas et al., 2014). In fact, elicitors mimic the effects of plant signal molecules and produce reactive oxygen species (ROS), which consequently produce defense hormones, enzymatic and non-enzymatic antioxidants to reduce the effects of ROS mechanisms (Ruiz-García and Gómez-Plaza, 2013; Abdul Malik et al., 2020).

Thus, the use of biological or non-biological elicitors as product stimulators is a promising approach to obtain high product concentration and increased volumetric productivity in comparatively less time (Smetanska, 2008; Liu et al., 2019). However, the choice of an appropriate elicitor or a combination of elicitors is crucial to produce a particular set of secondary metabolites on a large scale (Ahmad et al., 2017). Abiotic elicitors are physical and chemical stresses which may include metal ions, inorganic compounds, ultraviolet radiation, or electric current (He et al., 2018). In contrast, biotic elicitors are the substances which have biological origin, such as polysaccharides of plant cell walls (gum, pectic acid, cellulose) and components of microbial cell walls (chitin, glucans, chitosan) (Goyal et al., 2012). Therefore, elicitation of secondary metabolites by the activation of stress signaling factors might be an effective strategy.

Methyl jasmonate (MeJA) is a significant signaling molecule which enables plants to respond to injury or invasion by the pathogens (Yang et al., 2015; Hall et al., 2020). Under *in vitro* conditions, it induces the production of various plant secondary metabolites such as indole terpenoids, alkaloids, and rosmarinic acid (Smetanska, 2008). Various studies revealed the effect of MeJA on the amassing of secondary metabolites and antioxidants (Ali et al., 2015). Fungal elicitors, the fragments of fungal cell wall, have been used in various studies to boost secondary metabolite production in plants (Naik and Al-Khayri, 2016). According to different studies, secondary metabolites including ajmalicine, indole alkaloids, catharanthine, and serpentine were five times overproduced in cell suspensions of *Catharanthus roseus* (Moreno et al., 1993; Rijhwani and Shanks, 1998; Liang et al., 2018), while cultures of *Rauwolfiacanescens* also exhibited a significant rise in 12–oxophytodienoic acid and raucaffrincine.

Nanoparticles-induced oxidative stress can also be employed to induce medicinally important secondary metabolites production and accumulation in the plants (Fazal et al., 2016; Naik and Al-Khayri, 2016). Nanoparticles (NPs) are known to induce oxidative stress and act as effective elicitors which, in turn, results in overproduction of secondary metabolites under *in vitro* conditions (Tee et al., 2016; Fazal et al., 2019). The use of NPs is one of the most effective strategies for obtaining higher amounts of secondary metabolites as they also reduce the harmful effects of abiotic stress on plants (Jampílek and Král’ová, 2019; Xiao et al., 2019; Adrees et al., 2020; Linh et al., 2020). Various studies have revealed the accumulation of valuable secondary metabolites in culture medium due to abiotic stress induced by NPs as elicitors (Ramirez-Estrada et al., 2016; Jasim et al., 2017; Paramo et al., 2020). Therefore, it is a promising approach as it offers an alternative of using other chemicals having ecotoxic effects (Jasim et al., 2017; Javed et al., 2018; Mosavat et al., 2019). Silver nanoparticles (AgNPs) are among the top ten types of nanoparticles produced all over world as they possess antimicrobial, phytotoxic and cytotoxic properties (Nel et al., 2006; Keller et al., 2013). Different studies indicate the accumulation and biosynthesis of secondary metabolites including phenolic compounds (Chung et al., 2018), phytoalexins (hydroxycamalexin O-hexoside, camalexin, and hydroxylcamalexinmalonyl-hexoside) (Kruszka et al., 2020; Rivero-Montejo et al., 2021), Chicoric acid (Ramezannezhad et al., 2019), and flavonoids (Khan et al., 2021) in the presence of AgNPs.

Hydroponics is a modern agricultural system of soilless crops where plants are grown in a nutrient-rich solution with sufficient dissolved oxygen (Roberto, 2003; Liu et al., 2019). It is a technique of soilless cultivation in which the roots, anchored by growth substrates such as rocks, clay or perlite, are suspended in water supplemented with mineral nutrients (Maucieri et al., 2019; Lee et al., 2020). In order to maximize plant yield, a careful and regular monitoring is needed to optimize the composition of nutrient solution (Jung, 2004; Kumar and Cho, 2014).

Keeping in view the medicinal value of *S. marianum* and issues related to its low yield due to various factors, the current study was designed to establish hydroponic culture system and stimulate pharmacologically important secondary metabolites in response to different elicitors, like MeJA, AgNPs, and fungal elicitors. The elicitors used both individually and in combination, to investigate the amount of secondary metabolites production under *in vitro* conditions. The combined effect of these elicitors has not been studied previously in the hydroponic system.

## Materials and methods

### Sterilization of seeds and preparation of sand

Seeds of *Silybum marianum* were obtained from National Agriculture Research Centre (NARC) Islamabad, Pakistan. The seeds were first rinsed with distilled water and then washed with 70% ethanol (C_2_H_5_OH) for 1 min. The seeds already washed with distilled water were treated with ethanol, followed by three times washing with distilled water to eliminate the traces of ethanol. Then, 0.01% mercuric chloride solution was used for 2 min followed by four to five times washing with distilled water (Ramakrishna et al., 1991; Ahmad et al., 2016). The sand was prepared by treating already washed sand with sodium hypochlorite for 5 min, followed by washing again for 3–5 times with distilled water to exterminate the residues of sodium hypochlorite. Sand was then placed in oven at 70°C for drying.

### Seed germination and shifting to hydroponic system

The seeds were sown in sterile sand under controlled conditions in climate control room (24–25°C, 20–700 μmol m^–2^s^–1^). After germination, the seedlings were regularly watered and supplemented with Hoagland solution containing macronutrients [Ca(No_3_)_2_.4H_2_O, KNO_3_, KH_2_PO_4_, MgSO_4_.7H_2_O], micronutrients (H_3_BO_3_, MgCl_2_.4H_2_O, ZnSO_4_.7H_2_O, CuSO_4_.5H_2_O, and NaMoO_4_) and trace elements essential for plant growth (Hoagland and Arnon, 1950). Initially, six seedlings were sown in each pot that were thinned to one seedling per pot and transplanted to hydroponic system after 15 days of germination (Walters and Currey, 2018).

### Hydroponic culture

Hydroponic system was used following the protocol of Hoagland and Arnon (1938) with slight modifications. For the hydroponic system, a plant holder was made using foam with a hole plugged with the autoclaved cotton plug. The foam panel was cut a little smaller than the size of container. The hydroponic container was then filled with Hoagland solution. The seedlings were transplanted and an air-pump system was installed in the system in order to ensure proper aeration (Nguyen et al., 2016). The seedlings were then allowed to grow in seven different treatments and one was set as control (Table 1). The control was supplemented with Hoagland solution only, while different elicitors were used for the experimental cultures. The experiment was performed in Complete Randomized Design (CRD) using five replicates. For this purpose, 30 seedlings were set in hydroponics system for each treatment. To be specific, 6 harvests were carried out for each treatment and 5 seedlings were grown for each harvest. First harvest was done after 24 h of treatment, second harvest was done after 48 h of treatment, third harvest was done after 72 h of treatment, fourth harvest was done after 96 h of treatment, fifth harvest was done after 120 h of treatment, and finally sixth harvest was done after 144 h of treatment. The treatment time was of 6 days with an interval of 24 h among each harvest.

**TABLE 1:** Different treatments with tags.

No.	Treatments	Concentrations	Tags
1	Control	No treatment	t1
2	MeJA	100 μM	t2
3	Fungal elicitors	200 mg/L	t3
4	Green synthesized AgNPs	1 ppm	t4
5	MeJA + Fungal elicitors	100 μM + 200 mg/L	t5
6	MeJA + Green synthesized AgNPs	100 μM + 1 ppm	t6
7	Fungal elicitors + Green synthesized AgNPs	200 mg/L + 1 ppm	t7
8	Fungal elicitors + Green synthesized AgNPs + MeJA	200 mg/L + 1 ppm + 100 μM	t8

### Elicitors and their composition

Different elicitors were used in the experiment to induce the production of secondary metabolites. For this purpose, methyl jasmonate (Sigma-Aldrich) (100 μM), fungal elicitors (200 mg/L) prepared as described in the literature (Gong et al., 2018) and sonicated green synthesized AgNPs (1 ppm) were added, individually and in combination, in the hydroponic medium. The green synthesis of AgNPs was done by the reaction of AgNO_3_ and extract obtained from *Fortunella margarita* as described by Al-Kalifawi et al. (2015), with slight modifications in temperature and pH conditions. For all the hydroponic treatments, Hoagland’s solution was pre-added in the containers. The combinations of elicitors used as different treatments are shown in Table 1. The plants were kept under controlled conditions at 24–25°C.

### Phytochemical analyses

Plants were harvested after every 24 h and washed off. First harvest was done after 24 h followed by 48 h, 72 h, 96 h, 120 h, and 144 h of treatment. After harvesting, plants were subjected to phytochemical analyses after recording their fresh weight in grams. Plant leaves were used for various phytochemical analyses which included total flavonoid content (μg g^–1^ FW), total phenolic content (μg g^–1^ FW), determination of nitric oxide (nmol g^–1^ FW), and estimation of superoxide dismutase (Ug^–1^ FW).

#### Total flavonoid content

Total flavonoid content was measured following the protocol of Zhishen et al. (1999). Extract was placed in a 10 mL volumetric flask. Distilled water was added to make 5 mL, and 0.3 mL sodium nitrite (NaNO_2_) (1:20 v/v), 0.3 mL aluminum chloride (AlCl_3_) (1:10 v/v) were added 5 min later. After 6 min, 2 mL of 1 M sodium hydroxide (NaOH) was added keeping the total volume up to 10 mL with distilled water. The solution was mixed well again and the absorbance was measured against a blank at 510 nm by a spectrophotometer. The calibration curve was used to determine flavonoid content and expressed as percentage of quercetin (C_15_H_10_O_7_) in the extract (Eberhardt et al., 2000).

#### Total phenolic content

Total phenol content was calculated by bringing slight modifications in Folin-Ciocalteu method (Wolfe et al., 2003). The extract was mixed with 5 mL of Folin-Ciocalteu reagent (previously diluted to 1:10 *v/v* with water) and 4 mL (75 g/L) of sodium carbonate (Na2CO3). The mixture was vortexed for 15 s and incubated at 40°C for 30 min to develop color. The absorbance was read at 765 nm and total phenol content was expressed in mg/g of tannic acid (C_76_H_52_O_46_) equivalent using the following equation based on calibration curve: *y* = 0.1216x, *r*^2^ = 0.9365, where x is absorbance and y is tannic acid equivalent (mg/g).

#### Determination of nitric oxide

Nitric oxide (NO) is produced by different types of cells in picomolar to nanomolar range, and has a very short half-life in biological fluids (t1/2 < 5s). It is very difficult to directly measure its production and, therefore, nitrite (NO_2_^–^) and nitrate (NO_3_^–^) contents are determined as they are the stable products of NO oxidation. For this reason, NO_2_^–^ and NO^3–^ are often used to estimate the levels of NO in biological fluids and cell culture medium (Muller). The concentration of nitrite is generally measured by a well-known method, i.e., Griess colorimetric test (Hu et al., 2019). According to this method, Griess reagent (C_12_H_14_N_2_ + C_6_H_8_N_2_O_2_S + H_3_PO_4_) (100 μL), nitrite (NO_2_^–^) containing sample (300 μL) and deionized water (2.6 mL) were mixed in the spectrophotometer cuvette. The mixture was then incubated at room temperature for 30 min. A photometric reference sample was prepared by mixing 100 μL of Griess reagent and 2.9 mL of deionized water. The absorbance of nitrite containing sample was measured at 548 nm relative to the reference sample. Finally, the absorbance value was compared with a standard curve generated from the known NO.

#### Superoxide dismutase activity

The modified method by Kakkar et al. (1984) was used to determine the activity of SOD. The assay mixture contained 0.1 mL of plant sample, 1.2 mL of sodium phosphate buffer (pH 8.3, 0.052 M), 0.1 mL of phenazinemethosulphate (C_14_H_14_N_2_O_4_S) (186 μM), 0.3 mL of nitro blue tetrazolium (C_40_H_30_Cl_2_N_10_O_6_) (300 μM) and 0.2 mL of NADH (750 μM). The reaction was initiated by the addition of NADH. After incubation at 30°C for 90 s, the reaction was stopped by adding glacial acetic acid (0.1 mL). Then after adding 4 mL of *n*-butanol (C4H10O), the reaction mixture was agitated vigorously and incubated at room temperature for 10 min. After 10 min, the mixture was centrifuged and the butanol layer was separated. The absorbance of butanol layer was measured (560 nm) and the concentration of SOD was expressed as units/g of plant tissue. Then, the absorbance values were compared with a standard curve generated from known SOD.

#### Statistical analysis

The data collected was subjected to analysis of variance (ANOVA) and Scheffe *post hoc* test using repeated measurement with the help of statistical software SPSS version 2025. The difference, among means of treatments, was determined using least significant difference (LSD) at 5% significance level.

## Results

**FIGURE 1:**
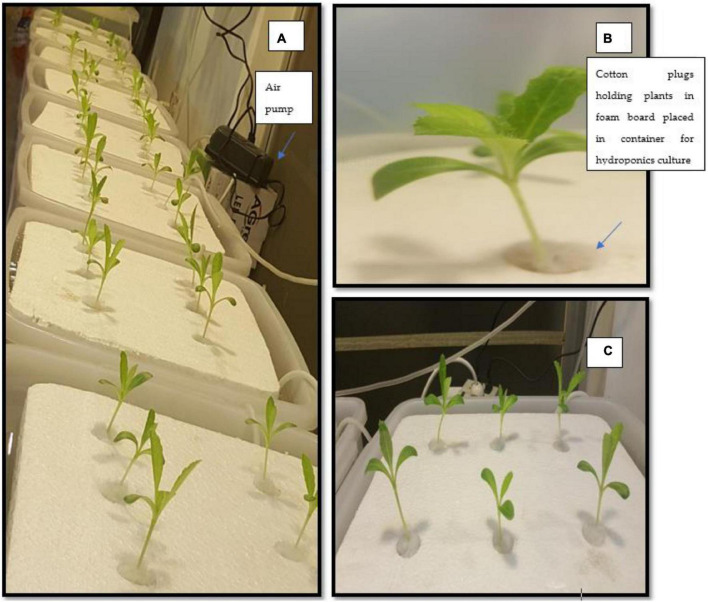
Hydroponically grown seedlings of *Silybum marianum*.

In the current study *S. marianum* was hydroponically grown to investigate the effect of different elicitors on production of secondary metabolites (Figures 1A–C).

The results indicated a substantial rise in secondary metabolite production in response to the elicitors used in the study (Tables 2, 5, 8, 11). Pairwise comparisons of treatment by treatment (Tables 3, 6, 9, 12) and harvest by harvest (Tables 4, 7, 10, 13) were also conducted using Scheffe *post hoc* test as done in previous studies (Nunes et al., 2011; Jacotot et al., 2019; Pérez-Castiñeira et al., 2021).

**TABLE 2:** Flavonoid content (μg g^–1^) of *Silybum marianum* exposed to different treatments of elicitors.

	24 h harvest	48 h harvest	72 h harvest	96 h harvest	120 h harvest	144 h harvest
Treatments	Mean ± S.D	Mean ± S.D	Mean ± S.D	Mean ± S.D	Mean ± S.D	Mean ± S.D
t1	284 ± 0.4	18 ± 0.4	224 ± 0.4	184 ± 0.4	135 ± 0.4	103 ± 0.4
t2	180 ± 0.4	153 ± 0.5	304 ± 0.3	195 ± 0.3	164 ± 0.4	165 ± 0.4
t3	20.2 ± 0.4	193 ± 0.4	217 ± 0.4	226 ± 0.4	109 ± 0.3	154 ± 0.4
t4	22.9 ± 0.4	245 ± 0.4	217 ± 0.4	192 ± 0.4	113 ± 2.5	168 ± 0.4
t5	13.5 ± 0.4	164 ± 0.4	215 ± 0.4	210 ± 0.4	150 ± 0.3	117 ± 0.5
t6	18.1 ± 0.4	197 ± 0.4	121 ± 0.4	180 ± 0.4	180 ± 0.5	165 ± 0.4
t7	12.5 ± 0.4	215 ± 0.4	214 ± 0.4	135 ± 0.3	150 ± 0.5	7.7 ± 0.4
t8	12 ± 0.4	151 ± 0.4	169 ± 0.4	7.8 ± 0.3	152 ± 0.3	129 ± 10.7

The values were recorded using least significant difference (LSD) at 5% significance level. t1 = control, t2 = methyl jasmonate, t3 = silver nanoparticles, t4 = fungal elicitors, t5 = methyl jasmonate + fungal elicitors, t6 = methyl jasmonate + silver nanoparticles, t7 = fungal elicitors + Silver nanoparticles, t8 = methyl jasmonate + silver nanoparticles + fungal elicitors.

**TABLE 3:** Pairwise comparison of the treatments for flavonoids by Scheffe *post hoc* test.

	t1	t2	t3	t4	t5	t6	t7	t8
t1	−	0.798	1.000	0.778	0.021	0.21	0.000*	0.000*
t2	0.798	−	0.643	1.00	0.000*	0.004*	0.000*	0.000*
t3	1.000	0.643	−	0.61	0.042	0.33	0.000*	0.000*
t4	0.778	1.000	0.619	−	0.000*	0.004*	0.000*	0.000*
t5	0.021	0.000*	0.042	0.000*	−	0.97	0.36	0.000*
t6	0.211	0.004*	0.335	0.004*	0.97	−	0.04	0.000*
t7	0.000*	0.000*	0.000*	0.000*	0.365	0.048	−	0.028
t8	0.000*	0.000*	0.000*	0.000*	0.000*	0.000*	0.028	−

*Significance at the 0.05 level.

**TABLE 4:** Pairwise comparison of the harvests for flavonoids by Scheffe *post hoc* test.

	24 h harvest	48 h harvest	72 h harvest	96 h harvest	120 h harvest	144 h harvest
24 h harvest	−	0.000*	0.000*	0.000*	0.000*	0.000*
48 h harvest	0.000*	−	0.000*	0.000*	0.000*	0.000*
72 h harvest	0.000*	0.000*	−	0.000*	0.000*	0.000*
96 h harvest	0.000*	0.000*	0.000*	−	0.000*	0.000*
120 h harvest	0.000*	0.000*	0.000*	0.000*	−	1.000
144 h harvest	0.000*	0.000*	0.000*	0.000*	1.000	−

*Significance at the 0.05 level.

**TABLE 5:** Total phenolic content (μg g^–1^) of *Silybum marianum* exposed to different treatments of elicitors.

	24 h harvest	48 h harvest	72 h harvest	96 h harvest	120 h harvest	144 h harvest
Treatments	Mean ± S.D	Mean ± S.D	Mean ± S.D	Mean ± S.D	Mean ± S.D	Mean ± S.D
t1	257.6 ± 2.1	152 ± 2.9	305.3 ± 2.6	321.5 ± 1.9	247.8 ± 2.5	285.1 ± 2.5
t2	348.2 ± 2.8	110.9 ± 2.9	332.0 ± 5.9	146.9 ± 2.5	372.0 ± 2.4	127.6 ± 2.5
t3	258.7 ± 2.5	221.1 ± 2.6	231.5 ± 2.4	175.5 ± 2.9	355.5 ± 2.03	228.4 ± 2.6
t4	274.5 ± 2.8	282.2 ± 2.5	242.9 ± 1.7	182.9 ± 2.6	175.8 ± 2.2	431.8 ± 2.5
t5	347.5 ± 2.6	127.3 ± 2.9	194.0 ± 4.7	295.3 ± 80.4	397.3 ± 3.5	357.1 ± 2.07
t6	340 ± 2.9	121.5 ± 2.5	229.1 ± 2.9	193.3 ± 1.5	265.6 ± 2.6	294.2 ± 2.5
t7	128.7 ± 2.2	268.9 ± 2.6	168.7 ± 2.1	167.1 ± 3.2	306.7 ± 2.1	236.9 ± 2.2
t8	129.6 ± 2.2	231.5 ± 2.1	266.4 ± 2.8	220 ± 2.03	227.6 ± 2.5	290.9 ± 2.9

The values were recorded using least significant difference (LSD) at 5% significance level. t1 = control, t2 = methyl jasmonate, t3 = silver nanoparticles, t4 = fungal elicitors, t5 = methyl jasmonate + fungal elicitors, t6 = methyl jasmonate + silver nanoparticles, t7 = fungal elicitors + Silver nanoparticles, t8 = methyl jasmonate + silver nanoparticles + fungal elicitors.

**TABLE 6:** Pairwise comparison of the treatments for total phenolics by Scheffe *post hoc* test.

	t1	t2	t3	t4	t5	t6	t7	t8
t1	−	0.002*	0.037	0.997	0.000*	0.003*	0.000*	0.000*
t2	0.002*	−	0.959	0.000*	0.000*	1.000	0.000*	0.278
t3	0.037	0.959	−	0.005*	0.000*	0.987	0.000*	0.022
t4	0.997	0.000*	0.005*	−	0.002*	0.000*	0.000*	0.000*
t5	0.000*	0.000*	0.000*	0.002*	−	0.000*	0.000*	0.000*
t6	0.003*	1.000	0.987	0.000*	0.000*	−	0.000*	0.191
t7	0.000*	0.000*	0.000*	0.000*	0.000*	0.000*	−	0.084
t8	0.000*	0.278	0.022	0.000*	0.000*	0.191	0.084	−

*Significance at the 0.05 level.

**TABLE 7:** Pairwise comparison of the harvests for total phenolics by Scheffe *post hoc* test.

	24 h harvest	48 h harvest	72 h harvest	96 h harvest	120 h harvest	144 h harvest
24 h harvest	−	0.000*	0.000*	0.000*	0.000*	0.000*
48 h harvest	0.000*	−	0.000*	0.001*	0.000*	0.000*
72 h harvest	0.000*	0.000*	−	0.000*	0.000*	0.000*
96 h harvest	0.000*	0.001*	0.000*	−	0.000*	0.000*
120 h harvest	0.000*	0.000*	0.000*	0.000*	−	0.000*
144 h harvest	0.000*	0.000*	0.000*	0.000*	0.000*	−

*Significance at 0.05 level.

**TABLE 8:** NO levels (nmole g^–1^) of *Silybum marianum* exposed to different treatments of elicitors.

	24 h harvest	48 h harvest	72 h harvest	96 h harvest	120 h harvest	144 h harvest
Treatments	Mean ± S.D	Mean ± S.D	Mean ± S.D	Mean ± S.D	Mean ± S.D	Mean ± S.D
t1	29.3 ± 0.87	37.1 ± 0.7	39.5 ± 0.7	35 ± 0.6	42.1 ± 0.8	39.4 ± 0.7
t2	33 ± 0.81	39.5 ± 0.7	54.2 ± 0.8	63.5 ± 0.7	35.4 ± 0.7	36.7 ± 0.7
t3	28.6 ± 0.5	43.7 ± 9.8	56.4 ± 10.3	38.4 ± 2.9	43 ± 1.5	40.4 ± 3.2
t4	40.8 ± 12.5	63.9 ± 0.8	62.7 ± 1.0	64.5 ± 1.2	57.3 ± 11.8	42.3 ± 11.7
t5	63.1 ± 2.1	54.3 ± 13.3	31.7 ± 0.4	31.9 ± 3.3	38.3 ± 1.01	37.7 ± 4.8
t6	31.4 ± 5.7	41.4 ± 2.5	36.9 ± 0.6	38 ± 1.04	198.6 ± 0.5	197.2 ± 1.4
t7	32.3 ± 1.8	29.5 ± 1.4	32.7 ± 1.9	24.1 ± 15.3	44 ± 2.6	44.2 ± 9.5
t8	54.8 ± 14.3	32.6 ± 3.9	39 ± 3.6	34.7 ± 3.2	39.9 ± 0.9	39.9 ± 0.7

The values were recorded using least significant difference (LSD) at 5% significance level. t1 = control, t2 = methyl jasmonate, t3 = silver nanoparticles, t4 = fungal elicitors, t5 = methyl jasmonate + fungal elicitors, t6 = methyl jasmonate + silver nanoparticles, t7 = fungal elicitors + Silver nanoparticles, t8 = methyl jasmonate + silver nanoparticles + fungal elicitors.

**TABLE 9:** Pairwise comparison of the treatments for NO by Scheffe *post hoc* test.

	t1	t2	t3	t4	t5	t6	t7	t8
t1	−	0.001*	0.054	0.000*	0.007	0.000*	0.666	0.462
t2	0.001*	−	0.888	0.000*	0.999	0.000*	0.000*	0.265
t3	0.054	0.888	−	0.000*	0.996	0.000	0.000	0.963
t4	0.000*	0.000*	0.000*	−	0.000*	0.000*	0.000*	0.000*
t5	0.007	0.999	0.996	0.000*	−	0.000*	0.000*	0.631
t6	0.000*	0.000*	0.000*	0.000*	0.000*	−	0.000*	0.000*
t7	0.666	0.000*	0.000*	0.000*	0.000*	0.000*	−	0.009
t8	0.462	0.265	0.963	0.000*	0.631	0.000*	0.009	−

*Significance at 0.05 level.

**TABLE 10:** Pairwise comparison of the harvests for NO by Scheffe *post hoc* test.

	24 h harvest	48 h harvest	72 h harvest	96 h harvest	120 h harvest	144 h harvest
24 h harvest	−	0.484	0.002*	1.000	0.000*	0.000*
48 h harvest	0.484	−	1.000	1.000	0.000*	0.000*
72 h harvest	0.002*	1.000	−	0.283	0.000*	0.000*
96 h harvest	1.000	1.000	0.283	−	0.000*	0.000*
120 h harvest	0.000*	0.000*	0.000*	0.000*	−	1.000
144 h harvest	0.000*	0.000*	0.000*	0.000*	1.000	−

*Significance at 0.05 level.

**TABLE 11:** SOD levels (U g^–1^) of *Silybum marianum* exposed to different treatments of elicitors.

	24 h harvest	48 h harvest	72 h harvest	96 h harvest	120 h harvest	144 h harvest
Treatments	Mean ± S.D	Mean ± S.D	Mean ± S.D	Mean ± S.D	Mean ± S.D	Mean ± S.D
t1	6.74 ± 0.3	15 ± 0.6	12.9 ± 0.5	14.3 ± 0.5	11.4 ± 0.3	14.8 ± 0.4
t2	11.6 ± 0.6	14.6 ± 0.5	14.9 ± 0.4	16.2 ± 0.3	14.5 ± 0.7	14.6 ± 0.6
t3	12.9 ± 0.4	9.6 ± 0.3	12.6 ± 0.5	14.3 ± 0.5	15.3 ± 0.5	9.9 ± 0.4
t4	11.5 ± 0.4	15.8 ± 0.4	13.1 ± 0.4	14.4 ± 0.6	11.9 ± 0.3	5.9 ± 0.4
t5	5.8 ± 0.4	7.5 ± 0.4	14.5 ± 0.5	11.5 ± 4.7	14.2 ± 0.4	14.6 ± 0.7
t6	14.5 ± 0.5	14.8 ± 0.6	16.5 ± 0.4	13.9 ± 0.5	13.8 ± 0.6	16.2 ± 0.5
t7	17.8 ± 0.8	6.7 ± 0.8	14.5 ± 0.4	14.1 ± 0.6	14.4 ± 0.5	8.6 ± 0.4
t8	14.5 ± 0.4	5.2 ± 0.7	14.5 ± 0.8	15.6 ± 0.7	11.4 ± 0.4	2.2 ± 0.5

The values were recorded using least significant difference (LSD) at 5% significance level. t1 = control, t2 = methyl jasmonate, t3 = silver nanoparticles, t4 = fungal elicitors, t5 = methyl jasmonate + fungal elicitors, t6 = methyl jasmonate + silver nanoparticles, t7 = fungal elicitors + Silver nanoparticles, t8 = methyl jasmonate + silver nanoparticles + fungal elicitors.

**TABLE 12:** Pairwise comparison of the treatments for SOD by Scheffe *post hoc* test.

	t1	t2	t3	t4	t5	t6	t7	t8
t1	−	0.000*	0.999	0.593	0.006	0.000*	0.000*	0.000*
t2	0.000*	−	0.000*	0.000*	0.000*	0.620	0.000*	0.000*
t3	0.999	0.000*	−	0.901	0.028	0.000*	0.000*	0.000*
t4	0.593	0.000*	0.901	−	0.442	0.000*	0.000*	0.000*
t5	0.006	0.000*	0.028	0.442	−	0.000*	0.025	0.143
t6	0.000*	0.620	0.000*	0.000*	0.000*	−	0.000*	0.000*
t7	0.000*	0.000*	0.000*	0.000*	0.025	0.000*	−	0.997
t8	0.000*	0.000*	0.000*	0.000*	0.143	0.000*	0.997	−

*Significance at 0.05 level.

**TABLE 13:** Pairwise comparison of the harvests for SOD by Scheffe *post hoc* test.

	24 h harvest	48 h harvest	72 h harvest	96 h harvest	120 h harvest	144 h harvest
24 h harvest	−	0.000*	0.000*	0.000*	0.000*	0.000*
48 h harvest	0.000*	−	0.000*	0.000*	0.000*	0.105
72 h harvest	0.000*	0.000*	−	0.000*	0.000*	0.000*
96 h harvest	0.000*	0.000*	0.000*	−	0.032	0.000*
120 h harvest	0.000*	0.000*	0.000*	0.032	−	0.000*
144 h harvest	0.000*	0.105	0.000*	0.000*	0.000*	−

*Significance at 0.05 level.

### Flavonoids levels in treated plants

**FIGURE 2:**
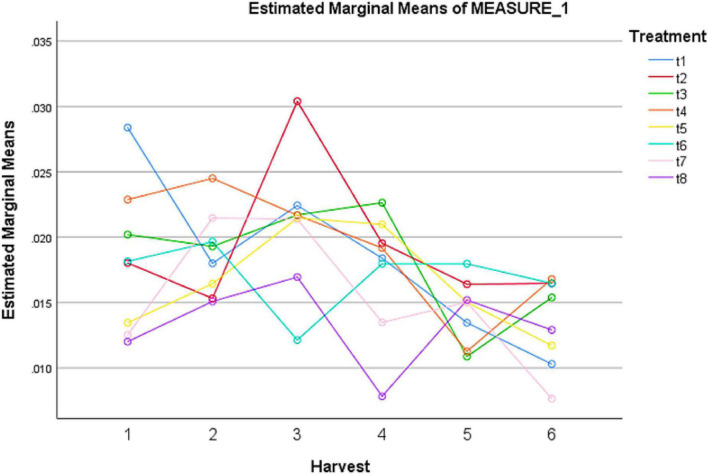
Effect of different treatments of elicitors on flavonoid content of *Silybum marianum* in hydroponics. The harvest time is on *x*-axis; 1 = 24 h, 2 = 48 h, 3 = 72 h, 4 = 96 h, 5 = 120 h, 6 = 144 h. For different treatments, t1 = control, t2 = methyl jasmonate, t3 = silver nanoparticles, t4 = fungal elicitors, t5 = methyl jasmonate + fungal elicitors, t6 = methyl jasmonate + silver nanoparticles, t7 = fungal elicitors + Silver nanoparticles, t8 = methyl jasmonate + silver nanoparticles + fungal elicitors.

The effect of treatments of different elicitors used in the study showed significant effect on flavonoids contents (Table 2). The highest concentration of flavonoids was observed in the presence of MeJA (100 μM) after 72 h of treatment (Figure 2). The pairwise comparisons of treatments (Table 3) and harvests (Table 4) showed both significance and non-significance among them.

### Total phenolic content in treated plants

**FIGURE 3:**
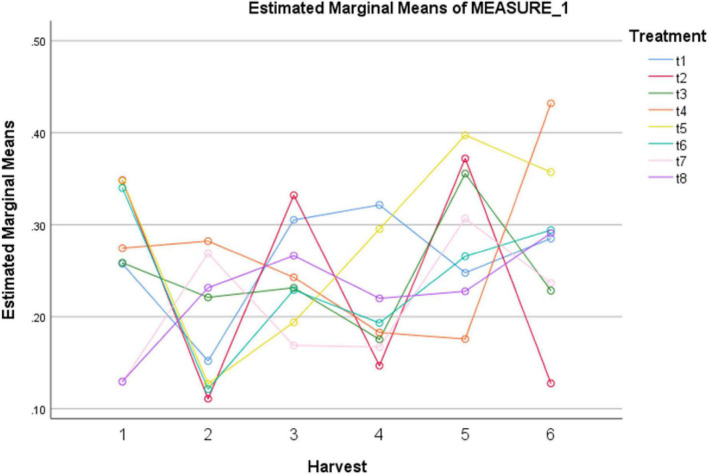
Effect of different treatments of elicitors on total phenolic content of *Silybum marianum* in hydroponics. The harvest time is on *x*-axis; 1 = 24 h, 2 = 48 h, 3 = 72 h, 4 = 96 h, 5 = 120 h, 6 = 144 h. For different treatments, t1 = control, t2 = methyl jasmonate, t3 = silver nanoparticles, t4 = fungal elicitors, t5 = methyl jasmonate + fungal elicitors, t6 = methyl jasmonate + silver nanoparticles, t7 = fungal elicitors + Silver nanoparticles, t8 = methyl jasmonate + silver nanoparticles + fungal elicitors.

In present study a notable rise in total phenolics levels was observed under different treatments as indicated in Table 5. Among different treatments, MeJA (100 μM), MeJA (100 μM) + fungal elicitors (0.2 g/L), and MeJA (100 μM) + AgNPs (1 ppm) application showed improved total phenolic content after 24 h. After 72 h, MeJA (100 μM) exhibited higher total phenolic content compared to other treatments. However, after 120 h, MeJA (100 μM) and MeJA (100 μM) + fungal elicitors (0.2 g/L) were the most effective in modulating total phenolics production. The highest total phenolic production was observed after 144 h under AgNPs treatment (1 ppm) (Figure 3). The pairwise comparisons of treatments (Table 6) and harvests (Table 7) showed both significance and non-significance among them.

### Nitric oxide levels in treated plants

**FIGURE 4:**
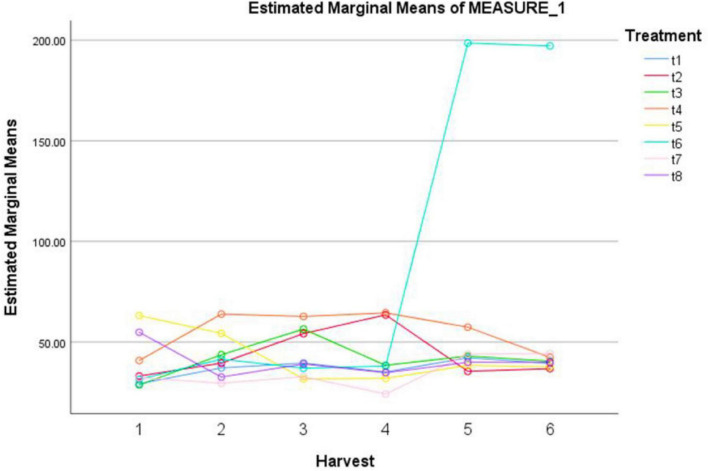
Effect of different treatments of elicitors on nitric oxide levels of *Silybum marianum* in hydroponics. The harvest time is on *x*-axis; 1 = 24 h, 2 = 48 h, 3 = 72 h, 4 = 96 h, 5 = 120 h, 6 = 144 h. For different treatments, t1 = control, t2 = methyl jasmonate, t3 = silver nanoparticles, t4 = fungal elicitors, t5 = methyl jasmonate + fungal elicitors, t6 = methyl jasmonate + silver nanoparticles, t7 = fungal elicitors + Silver nanoparticles, t8 = methyl jasmonate + silver nanoparticles + fungal elicitors.

In our study, the treatments with different elicitors showed a significant effect on NO activity (Table 8). The highest NO activity was observed under the combination of MeJA (100μM) + AgNPs (1 ppm) after 120 and 144 h (Figure 4). The pairwise comparisons of treatments (Table 9) and harvests (Table 10) showed both significance and non-significance among them.

### Superoxide dismutase levels in treated plants

**FIGURE 5:**
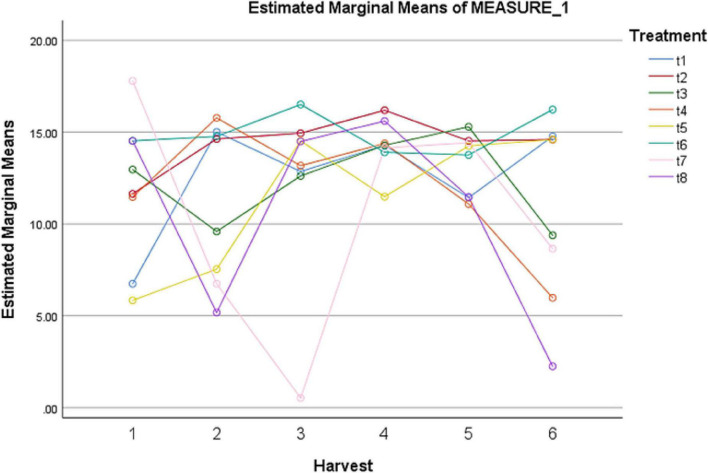
Effect of different treatments of elicitors on SOD levels of *Silybum marianum* in hydroponics. The harvest time is on *x*-axis; 1 = 24 h, 2 = 48 h, 3 = 72 h, 4 = 96 h, 5 = 120 h, 6 = 144 h. For different treatments, t1 = control, t2 = methyl jasmonate, t3 = silver nanoparticles, t4 = fungal elicitors, t5 = methyl jasmonate + fungal elicitors, t6 = methyl jasmonate + silver nanoparticles, t7 = fungal elicitors + Silver nanoparticles, t8 = methyl jasmonate + silver nanoparticles + fungal elicitors.

A remarkable effect of different elicitors was observed on SOD content (Table 11). A sharp increase in SOD concentration was observed after 24 h under fungal elicitors (0.2 g/L) + AgNPs (1 ppm). However, this treatment showed the lowest SOD levels after 72 h of treatment. Among other treatments, MeJA (100 μM) + AgNPs (1 ppm) application showed the highest concentration of SOD after 72 and 144 h (Figure 5). The pairwise comparisons of treatments (Table 12) and harvests (Table 13) showed both significance and non-significance among them.

## Discussion

Exogenously used elicitors modulate the expression of a number of genes during plant development both under normal and stress conditions (Horbowicz et al., 2011b; Donati et al., 2019; Wang et al., 2021). These act as signaling molecules in response to environmental challenges and are crucial in the overproduction of important secondary metabolites, including flavonoids (Akula and Ravishankar, 2011). Among different elicitors, MeJA, a signaling molecule, is known to induce plant defense response by the production of a variety of secondary metabolites (Vezzulli et al., 2007; Balbontín et al., 2018). It has been observed that jasmonates take part in oxidative and physiological responses in plants following an abiotic stress event. Due to these facts, MeJA remained the prime focus of research in viticulture during the past few years, primarily because of its role in the synthesis of phenolic compounds (Gómez-Plaza et al., 2017; Portu et al., 2018). Likewise, fungal elicitors and AgNPs can initiate plant defense response which, consequently, results in augmentation of total phenolic contents and provides certain level of protection against pathogenic attack (Polkowska-Kowalczyk et al., 2013; Goyal and Mattoo, 2014). In addition to these compounds, early plant defense activation events also include NO production (Wang and Wu, 2005), which typically occurs in plant cells in response to pathogen invasion (Beligni and Lamattina, 2001). Plant growth, development, and defense mechanisms are regulated by NO as it is cucial in regulating plant defense or stress responses (Delledonne et al., 1998). The molecular characteristics of NO, such as its small size, short half-life, and high diffusivity, make it an excellent intercellular and intracellular signaling molecule for plant defense responses. Furthermore, superoxide dismutase (SOD) is an antioxidant enzyme useful for physiological defense mechanisms in plants against free radicals and reactive oxygen species (ROS) in response to biotic and abiotic stresses (Stephenie et al., 2020). The role of elicitors in overproduction of these crucial compounds is now evident in different researches. It has been observed that MeJA (Vezzulli et al., 2007), fungal elicitors (Foissner et al., 2000), and abiotic stressors (Garcês et al., 2001) imitate infectious pathogens, causing the accumulation of significant plant metabolites (Xu et al., 2005).

Keeping in view the crucial role of elicitors in the synthesis of valuable plant metabolites, different types of elicitors were used in the current study. The results indicated a substantial rise in secondary metabolite production in response to the elicitors used in the study (Tables 2, 5, 8, 11). Pairwise comparisons of treatment by treatment (Tables 3, 6, 9, 12) and harvest by harvest (Tables 4, 7, 10, 13) were also conducted using Scheffe *post hoc* test as done in previous studies (Nunes et al., 2011; Jacotot et al., 2019; Pérez-Castiñeira et al., 2021). According to different studies, pairwise comparison is a useful method to determine relationships between pairs of means in group comparisons (Makkar, 2010; Nunes et al., 2011; Jacotot et al., 2019; Pérez-Castiñeira et al., 2021). The statistical analyses showed significant differences among treatments and harvest times. Our results, indicating increased flavonoid contents due to MeJA application, are in line with the studies conducted by Mohamed and Latif (2017). They found that the application of MeJA is effective in regulating quercetin acid and rutin contents that lead to enhanced flavonoids in all soybean genotypes. Since the accumulation of flavonoids contributes to the defense mechanism of plants (Dixon and Paiva, 1995), the application of MeJA could be effective in inducing stress response (Karami et al., 2013). MeJA can also regulate the expression of wound-induced flavonoid genes, thereby increasing the concentration of flavonoids (Tamari et al., 1995). Moreover, MeJA has the ability to regulate plant secondary metabolism by stimulating the accumulation of flavonoids, alkaloids and phenols (Yan et al., 2013). Its methyl ester and jasmonic acid are thought to play a central role in the synthesis of flavonoids and other secondary metabolites (Horbowicz et al., 2011a). A study conducted on blackberries also showed that the flavonoid content and the antioxidant activity of the plant were enhanced under MeJA treatment (Wang et al., 2008).

In our study, the increase in enzymatic antioxidant activity is in line with the results (Nojavan-Asghari and Norastehnia, 2006), who found that MeJA (50 and 100 μM) stimulates the production of several antioxidant enzymes. It appears that MeJA plays a unique role in oxidative stress transduction pathways by increasing the activity of SOD and POX. It is also reported that signal transduction by MeJA occurs at approximately 50 μmol and is inhibited at concentrations greater than 100 μmol (Batool et al., 2009). In strawberry leaves, MeJA alters the ratio of membrane fatty acids which mainly target the free radicals (Li et al., 1998). The goal is to repair the damage caused by ROS which includes enzymes such as SOD and non-enzymes such as ascorbic acid and glutathione (Li et al., 1998). In another report by Aftab et al. (2011) SOD activities in leaves increased due to MeJA treatment. They also mentioned that application of MeJA further enhanced the activity of all antioxidant enzymes in unstressed and stressed plants by complementing ROS scavenging mechanism. MeJA can induce the antioxidant defense activity of plants, thus eliminating the possible toxic effects of free radicals and making plants more stress resistant. MeJA is reported to attenuate the ROS effect of strawberry and paraquat on corn seedlings underdrought stress (Choudhury and Panda, 2004).

Ulloa-Inostroza et al. (2019) also support our findings which showed that MeJA administration reduced oxidative damage through SOD. MeJA generally increases the concentration of SOD because it induces stress and hydrogen peroxide in plants (Soares et al., 2010). According to different studies, MeJA can induce hydrogen peroxide and SOD more effectively at low concentrations compared to high concentrations. Moreover, SOD levels are further increased when MeJA is applied directly to the leaves of *Brassica napus* grown under light and dark conditions (Comparot et al., 2002; Maksymiec and Krupa, 2002).

Our results showed that the application of MeJA increased plant NO content in accordance with Wang and Wu (2005), who explored the involvement of NO in secondary metabolic activity and defense response induced by MeJA. Their results showed MeJA-induced NO burst and its relationship to the number and timing of other defense responses in *Taxuschinensis* cell cultures. The kinetics of NO production induced by MeJA is similar to the kinetics induced by fungal elicitors in tobacco leaves and in tissues of *Taxusbrevifolia* (Foissner et al., 2000; Pedroso et al., 2000). Therefore, the production of NO is an early event that triggers plant defense responses. In addition to this, MeJA-induced NO production in yew cell cultures was increased by NO generators and inhibited by NO inhibitors and NO scavengers. In other plant species, NO donors and inhibitors have similar effects on the production of NO induced by microbial pathogens and mechanical stress (Delledonne et al., 1998; Garcês et al., 2001). Garcês et al. (2001) observed the maximum NO scavenging potential of cell extracts in MeJA treated plants. In our study, plants treated with MeJA showed a higher concentration of nitric oxide which is also evident with the fact that NO also acts as a stressor and causes the production of H_2_O_2_; thereby increasing NO levels (Wang and Wu, 2005). A study conducted on blueberries explained that H_2_O_2_ produced by NO further induces the pathway of phenylalanine and defense-related enzymes, thereby conferring disease resistance in plants (Wang et al., 2020). Therefore, NO is a very good ROS scavenger, which can reduce stress, induced by H_2_O_2_ in plants stimulated by MeJA (Hung and Kao, 2004).

Different concentrations of AgNPs used on *Brassica napus* have shown increased plant growth (Sharma et al., 2012). In the current study, AgNPs showed an increase in the concentration of plant metabolites including SOD which is consistent with the findings of Bagherzadeh Homaee and Ehsanpour (2016), where AgNP induced stress conditions in plants to synthesize SOD. AgNPs are known to modify the antioxidant system, thereby accumulating SOD and other antioxidant enzymes (Jiang et al., 2014). Another study conducted on red blood cells also proved that AgNPs affect the antioxidant system and thus induce H_2_O_2_ and SOD production (Fang et al., 2019). Lin and Xing (2007) also explained that AgNPs had a positive effect on silicon maleate levels leading to a significant rise in secondary metabolite synthesis. The application of AgNPs on mustard seeds caused increase in seedling vigor, plant height, root, and shoot weight (Sharma et al., 2012). In fact, AgNPs play an important role in plant growth as they interfere with different pathways in plant cells and regulate secondary metabolites production (Vannini et al., 2013). Hasanloo et al. (2013) studied the effects of different amounts of fungal elicitors on the cultivation of hairy roots of *S. marianum*. The study showed an enhanced production of flavonoids and improved root growth compared to the control hydroponics (Hasanloo et al., 2013).

Therefore, *Aspergillus niger*, MeJA, and AgNPs are proven to elicit cellular stress response communications connected with biosynthesis of the plant secondary metabolites (Masarovic̀ová et al., 2010; Szõllõsi et al., 2020). From the results of this study, it can be concluded that the application of MeJA is the most effective method to improve the production of phenolic compounds and enzymatic antioxidants of *S*. *marianum*. MeJA + AgNPs and AgNPs + fungal elicitors also exhibited enhanced production of secondary metabolites and enzymatic antioxidants. Under the influence of fungal elicitors and AgNPs + MeJA, the content of total flavonoids was increased. These results provide clear evidence that the plant defense mechanism is stimulated in the presence of these elicitors which, in turn, result in the biosynthesis of medicinally important compounds from vegetative plant parts.

## Conclusion

It can be concluded from the results of the present study that MeJA application is the most effective treatment to enhance the production of total phenolics and SOD activity in *S*. *marianum* followed by MeJA, MeJA + AgNPs, AgNPs, and fungal elicitors with improved secondary metabolites production and increased enzymatic antioxidants while, Increased total flavonoids were observed under the influence of fungal elicitors and MeJA + AgNPs application. We can also conclude that, treating *S. marianum* with elicitors in hydroponic culture could be a valuable tool to study the regulation of plants cells metabolism in response to different stress factors by increasing the metabolites production within short period of time.

### Recommendations

This technique is cheap and less laborious as compared to other biotechnological techniques to change plant’s response by modulating genetic expression and is therefore recommended that these elicitors should be apply on different plants which are grown under different culture mediums including tissue culture, sand culture, petri plate culture, soil culture, and hydroponics. Moreover, there are many types of nanoparticles with different sizes; therefore, using various culture techniques for application of these elicitors may produce extraordinary results.

## Data availability statement

The original contributions presented in this study are included in the article/supplementary material, further inquiries can be directed to the corresponding author.

## Author contributions

BM and AH: conceptualization, devised methodology, experimental strategy, validation, investigation, and writing—original draft. BM, RR, RM, and IU: formal analysis. AH: resources. AR, SE, MS, and TF had critically reviewed and revised the manuscript. All authors had participated in this study by working on different aspects including study design, statistical analysis, experimental work, write-up, finalization, and critical review.
